# A Separated Calibration Method for Inertial Measurement Units Mounted on Three-Axis Turntables

**DOI:** 10.3390/s18092846

**Published:** 2018-08-28

**Authors:** Chun-mei Dong, Shun-qing Ren, Xi-jun Chen, Zhen-huan Wang

**Affiliations:** Space Control and Inertial Technology Research Center, Harbin Institute of Technology, Harbin 150001, China; dcmjob@126.com (C.-m.D.); renshunqing@hit.edu.cn (S.-q.R.); zhenhuanwang@hit.edu.cn (Z.-h.W.)

**Keywords:** IMU, separated calibration, error modeling, calibration method, parameter estimation

## Abstract

Inertial Measurement Unit (IMU) calibration accuracy is easily affected by turntable errors, so the primary aim of this study is to reduce the dependence on the turntable’s precision during the calibration process. Firstly, the indicated-output of the IMU considering turntable errors is constructed and with the introduction of turntable errors, the functional relationship between turntable errors and the indicated-output was derived. Then, based on a D-suboptimal design, a calibration method for simultaneously identifying the IMU error model parameters and the turntable errors was proposed. Simulation results showed that some turntable errors could thus be effectively calibrated and automatically compensated. Finally, the theoretical validity was verified through experiments. Compared with the traditional method, the method proposed in this paper can significantly reduce the influence of the turntable errors on the IMU calibration accuracy.

## 1. Introduction

Strapdown inertial navigation systems (SINSs) are fully self-contained navigation systems that can continuously provide attitude, velocity and position information. They have been widely used in vehicles, missiles, aircrafts and ships. However, the errors of a SINS will accumulate and diverge with time, which will affect the navigation accuracy and restrict its combat capability to some extent. In order to improve the accuracy of a SINS, we must fully consider the characteristics of the system, and carry out corresponding research to solve structural design, error compensation, algorithm design, and calibration test issues, etc. [1,2,3,4].

The error of the inertial measurement unit (IMU) is one of the main sources of error for a SINS. The IMU mainly consists of inertial sensors such as accelerometers and gyros which measure the specific force and the angular rate of a vehicle, respectively, and output the navigation information through some algorithms [5,6]. Error compensation for SINS is an effective method to improve the accuracy of SINS navigation, whereas error calibration is a prerequisite for error compensation. Currently there are two types of error calibration method: systematic calibration and separated calibration. Systematic calibration uses the navigation output errors (such as attitude error, velocity error, and position error) as observations to identify the error parameters, the state vectors of parameters in the calibration model are estimated [7,8,9,10,11,12,13]. Because the navigation error is a synthesis of IMU errors, systematic calibration can reduce the dependence on turntable’s accuracy, but it has certain inadequacies, such as the fact that not all error parameters can be calibrated, and noise will impact the estimation accuracy [14,15]. Separated calibration is a classical method that has been used domestically and abroad. This method requires high-precision equipment (such as turntables) to provide the IMU with precise excitation of specific forces and angular rates [16], and the parameters in the calibration model can then be obtained via an optimal estimation algorithm [17,18,19,20]. The separated calibration method has the advantage of high precision, but its calibration accuracy for model parameters is easily affected by turntable errors and IMU mounting errors.

In the process of IMU calibration, the influence of turntable errors cannot be ignored. With the IMU being mounted onto a turntable, the IMU input axis lines are inevitably inclined relative to the inner axis lines of the turntable, thereby introducing mounting errors, so the practical specific forces and rates on the input axis lines of IMU are not the nominal values. These differences are called input errors. Studies for avoiding turntable errors in the calibration process mainly include the following aspects: In systematic calibration studies, reduction of turntable errors is mainly carried out using a rotational inertial navigation system (RINS). Since the research on rotating SINS has become a focus in recent years, rotation technology can be used as another method of reducing INS navigation errors. However, in a rotating mechanism, the use of turntables brings about additional errors reducing the navigation accuracy. In order to reduce the influence of the perpendicularity of the dual-axis rotational mechanism on calibration accuracy, a non-orthogonal angles mode [21] was established to estimate the perpendicularity, and then compensated for it, and thus the navigation accuracy of IMU was finally raised. In reference [22], the mounting errors between the IMU and rotational mechanism axes are introduced into the sensor model of the dual-axis rotational INS. The calibration method for mounting errors is designed using a thin-shell (TS) algorithm which is suitable for nonlinear large-angle error calibration and relies on known initial values. Subsequent data processing is time-consuming. It takes two steps to calibrate all the parameters, firstly the IMU errors are calibrated and compensated, then the mounting errors are estimated. In reference [23], a new self-calibration method for non-orthogonal angles in the tri-axis RINS is proposed.

In the study of separated calibration, to overcome the limitations of over-reliance on the turntable accuracy, the Dutch scholar Lötters proposed an accelerometer calibration method based on norm-observation in 1998 [24]. The proposed calibration methods based on norm-observation utilize the fact that ideally the norm of the measured output of the accelerometers and gyros are equal to the magnitude of applied force and rotational velocity, respectively [25,26,27]. In reference [28], the calibration of gyros is significantly improved by using the outputs of the IMU orientation integration algorithm, after arbitrary motions. The derivative properties of norm-observation allow the gyroscopes to be calibrated without external equipment, such as a turntable, or requiring precise maneuvers. In reference [29], the derivative properties of norm-observation together with a model of the sensors are used to construct a cost function, which is minimized with respect to the unknown model parameters using Newton’s method, requiring no mechanical platform for the accelerometer calibration and only a simple rotating table for the gyro calibration. We note that the calibration method based on norm-observation is universal, this method is applied in magnetometer calibration [30,31,32,33,34].

In summary, there are some problems of minimizing turntable errors in INS calibration. Firstly, in systematic calibration, though the non-orthogonal angles or installation errors between the turntable axes of the rotating mechanism for RINS can be calibrated, but cannot be identified at the same time. In addition, the parameters in the IMU error model are not fully observable. Finally, in separated calibration, though the norm-observation can reduce the precision requirements for the turntable, it cannot estimate all turntable errors. In this paper, a separated calibration method was adopted by introducing turntable errors into an IMU error calibration model which includes biases, scale factor errors, and installation errors of gyros and accelerometers in IMU and some turntable errors. Based on the D-suboptimal criterion, the calibration scheme was designed to simultaneously identify the IMU error parameters and the turntable errors, and automatically compensate for the turntable errors affecting IMU calibration accuracy.

## 2. The Establishment of the Error Model

### 2.1. Transformations between Frames

The definitions of the frames used in this study are shown in Table 1.

In this paper, the navigation frame directs east-north-up (ENU). Since the accelerometer and the gyro cannot be installed strictly orthogonally, *a* and *g* are not orthogonal frames. To convert the IMU outputs into elements under an orthogonal frame, the body frame is defined as follows: axis *x_b_* of the body frame coincides with the unit vector *x_a_* of X accelerometer’s sensitive axis, axis *y_b_* is perpendicular to *x_b_* in plane *x_a_y_a_*, while *y_a_* is the Y accelerometer’s sensitive axis, *z_b_*, *x_b_* and *y_b_* together form the right-hand right angle frame. With the turntable in the initial position where there is no errors, axes *x*, *y*, *z* of all the frames in Table 1 coincide and point to the east-north-up. The errors of the three-axis turntable include angular position errors, perpendicularities of adjacent axis lines, angular rate errors and zero position errors, etc. In this study, only perpendicularities and zero position errors are taken into account.

With the outer axis of the turntable turning to *γ*, the zero position error of the outer axis is represented by $\Delta \gamma_{0}$, and the 2-D perpendicularity of the outer axis versus the horizontal plane is represented by $\Delta \theta_{x0}$, $\Delta \theta_{y0}$. The attitude relationship between *t*_1_ and *n* is:

(1) $$C_{t1}^{n}=\left[\begin{array}{ccc} 1 & 0 & 0\\ 0 & 1 & -\Delta \theta_{x0}\\ 0 & \Delta \theta_{x0} & 1 \end{array}\right]\cdot \left[\begin{array}{ccc} 1 & 0 & \Delta \theta_{y0}\\ 0 & 1 & 0\\ -\Delta \theta_{y0} & 0 & 1 \end{array}\right]\cdot \left[\begin{array}{ccc} \cos(\gamma +\Delta \gamma_{0}) & -\sin(\gamma +\Delta \gamma_{0}) & 0\\ \sin(\gamma +\Delta \gamma_{0}) & \cos(\gamma +\Delta \gamma_{0}) & 0\\ 0 & 0 & 1 \end{array}\right]$$

With the middle axis of the turntable turning to *α*, the zero position error of the middle axis is represented by $\Delta \alpha_{0}$, and the perpendicularity between the middle and the outer axis lines is represented by $\Delta \theta_{om}$:

(2) $$C_{t2}^{t1}=\left[\begin{array}{ccc} 1 & 0 & \Delta \theta_{om}\\ 0 & 1 & 0\\ -\Delta \theta_{om} & 0 & 1 \end{array}\right]\cdot \left[\begin{array}{ccc} 1 & 0 & 0\\ 0 & \cos(\alpha +\Delta \alpha_{0}) & -\sin(\alpha +\Delta \alpha_{0})\\ 0 & \sin(\alpha +\Delta \alpha_{0}) & \cos(\alpha +\Delta \alpha_{0}) \end{array}\right]$$

With the inner axis of the turntable turning to β, the zero position error of the middle axis is represented by $\Delta \beta_{0}$, and the perpendicularity between the inner and the middle axis lines is represented by $\Delta \theta_{im}$:

(3) $$C_{t3}^{t2}=\left[\begin{array}{ccc} 1 & -\Delta \theta_{im} & 0\\ \Delta \theta_{im} & 1 & 0\\ 0 & 0 & 1 \end{array}\right]\cdot \left[\begin{array}{ccc} \cos(\beta +\Delta \beta_{0}) & 0 & \sin(\beta +\Delta \beta_{0})\\ 0 & 1 & 0\\ -\sin(\beta +\Delta \beta_{0}) & 0 & \cos(\beta +\Delta \beta_{0}) \end{array}\right]$$

Suppose the angular mounting errors between the frame of the turntable’s inner axis and the IMU body frame are Δ*η_x_*, Δ*η_y_*, Δ*η_z_*, then the attitude relationship between frames *b* and *t*_3_ is:

(4) $$C_{b}^{t3}=\left[\begin{array}{ccc} 1 & -\Delta \eta_{x} & \Delta \eta_{y}\\ \Delta \eta_{x} & 1 & -\Delta \eta_{z}\\ -\Delta \eta_{y} & \Delta \eta_{z} & 1 \end{array}\right]$$

Therefore, the attitude matrix between the IMU body frame and the navigation frame is:

(5) $$C_{n}^{b}={\left(C_{t1}^{n}C_{t2}^{t1}C_{t3}^{t2}C_{b}^{t3}\right)}^{T}$$

### 2.2. IMU Calibration Model on Three-Axis Turntable

Outputs of the accelerometers and the gyros can be expressed as:

(6) $$N_{a}=M_{a}\cdot f^{b}+b_{a}+\nu_{a}$$

(7) $$N_{g}=M_{g}\cdot \omega^{b}+b_{g}+\nu_{g}$$

where $N_{a}={\left[\begin{array}{ccc} N_{ax}/K_{ax} & N_{ay}/K_{ay} & N_{az}/K_{az} \end{array}\right]}^{T}$, $N_{ai}$ is the number of the accelerometer’s output pulses per unit time, $K_{ai}$ is the accelerometer’s scale factor; $N_{g}={\left[\begin{array}{ccc} N_{gx}/K_{gx} & N_{gy}/K_{gy} & N_{gz}/K_{gz} \end{array}\right]}^{T}$, $N_{gi}$ is the number of the gyro’s output pulses during time *t*, $K_{gi}$ is the gyro’s scale factor; $f^{b}$ and $\omega^{b}$ are the specific force vector and the angular rate vector with respect to the inertial space represented in the body frame *b*; $b_{a}={\left[\begin{array}{ccc} b_{ax} & b_{ay} & b_{ay} \end{array}\right]}^{T}$ and $b_{g}={\left[\begin{array}{ccc} b_{gx} & b_{gy} & b_{gy} \end{array}\right]}^{T}$ are the bias vectors of the accelerometers and the gyros respectively; $\nu_{a}$ and $\nu_{g}$ are the measurement noises of the accelerometers and the gyros:

$$M_{a}=\left[\begin{array}{ccc} 1+\Delta K_{ax} & 0 & 0\\ M_{ayx} & 1+\Delta K_{ay} & 0\\ M_{azx} & M_{azy} & 1+\Delta K_{az} \end{array}\right]$$

$$M_{g}=\left[\begin{array}{ccc} 1+\Delta K_{gx} & M_{gxy} & M_{gxz}\\ M_{gyx} & 1+\Delta K_{gy} & M_{gyz}\\ M_{gzx} & M_{gzy} & 1+\Delta K_{gz} \end{array}\right]$$

where $\Delta K_{ai}$ and $\Delta K_{gi}$ are the scale factor errors of the *i*th accelerometer and the *i*th gyro; $M_{aij}$ and $M_{gij}$ are the mounting errors, correspondingly.

Considering turntable errors, and Equations (1)–(5), with the accelerometers adopting a static position method on the turntable for calibration, the accelerometers’ input in the body frame can be expressed as:

(8) $$f^{b}=C_{n}^{b}\cdot {\left[\begin{array}{ccc} 0 & 0 & 1 \end{array}\right]}^{T}$$

When the gyro rotates 360° at the angular rate of $\omega_{T}$ around the outer axis of the turntable, inner axis and middle axis are at the static angular positions *α*, *β*, the gyro’s input in the body frame can be expressed as

(9) $$\omega^{b}=C_{n}^{b}\cdot {\left[\begin{array}{ccc} 0 & \omega_{e}\cos L & \omega_{e}\sin L \end{array}\right]}^{T}+C_{t1}^{b}\cdot {\left[\begin{array}{ccc} 0 & 0 & \omega_{T} \end{array}\right]}^{T}$$

If turntable errors are not considered, Equation (5) is represented as

$$C_{n}^{b}={\left(\left[\begin{array}{ccc} \cos\gamma & -\sin\gamma & 0\\ \sin\gamma & \cos\gamma & 0\\ 0 & 0 & 1 \end{array}\right]\left[\begin{array}{ccc} 1 & 0 & 0\\ 0 & \cos\alpha & -\sin\alpha \\ 0 & \sin\alpha & \cos\alpha \end{array}\right]\left[\begin{array}{ccc} \cos\beta & 0 & \sin\beta \\ 0 & 1 & 0\\ -\sin\beta & 0 & \cos\beta \end{array}\right]\right)}^{T}$$

Substituting the above equations into the error model of the accelerometer and the gyro, the traditional calibration model can be obtained

$$\left\{ \begin{array}{l} N_{x}{\;}^{a}=b_{ax}-(1+\Delta K_{ax})\cos\alpha \sin\beta \\ N_{y}{}^{a}=b_{ay}-M_{ayx}\cos\alpha \sin\beta +(1+\Delta K_{ay})\sin\alpha \\ N_{z}{}^{a}=b_{az}+(1+\Delta K_{az})\cos\alpha \cos\beta -M_{azx}\cos\alpha \sin\beta +M_{azy}\sin\alpha \end{array}\right.$$

and:

$$\left\{ \begin{array}{l} N_{x}{\;}^{g}=b_{gx}-(1+\Delta K_{gx})\cos\alpha \sin\beta +M_{gxy}\sin\alpha +M_{gxz}\cos\alpha \cos\beta \\ N_{y}{}^{g}=b_{gy}+(1+\Delta K_{gy})\sin\alpha -M_{gyx}\cos\alpha \sin\beta +M_{g}{}_{yz}\cos\alpha \cos\beta \\ N_{z}{}^{g}=b_{gz}+(1+\Delta K_{gz})\cos\alpha \cos\beta +M_{g}{}_{zy}\sin\alpha -M_{g}{}_{zx}\cos\alpha \sin\beta \end{array}\right.$$

Considering the turntable errors, combined with Equations (1)–(5), with the introduction of turntable errors into the accelerometer and gyro error model, that is, substituting Equations (8) and (9) into (6) and (7), and ignoring the second-order small quantity, a new calibration model is obtained:

(10) $$\left\{ \begin{array}{ll} N_{ax}= & b_{ax}-(1+\Delta K_{ax})\cos\alpha \sin\beta +\Delta \theta_{x0}(\sin\gamma \cos\beta +\cos\gamma \sin\alpha \sin\beta )+\Delta \theta_{y0}(-\cos\gamma \cos\beta +\sin\gamma \sin\alpha \sin\beta )\\ & +\Delta \eta_{z}\sin\alpha +\Delta \alpha_{0}\sin\alpha \sin\beta -(\Delta \beta_{0}+\Delta \eta_{y})\cos\alpha \cos\beta -\Delta \theta_{om}\cos\beta +\Delta \theta_{im}\sin\alpha \cos\beta \\ N_{ay}= & b_{ay}+(1+\Delta K_{ay})\sin\alpha +\Delta \theta_{x0}\cos\gamma \cos\alpha +\Delta \theta_{y0}\sin\gamma \cos\alpha \\ & +\Delta \alpha_{0}\cos\alpha +(\Delta \eta_{z}-M_{ayx})\cos\alpha \sin\beta +\Delta \eta_{x}\cos\alpha \cos\beta \\ N_{az}= & b_{az}+(1+\Delta K_{az})\cos\alpha \cos\beta +\Delta \theta_{x0}(\sin\gamma \sin\beta -\cos\gamma \sin\alpha \cos\beta )-\Delta \theta_{y0}(\cos\gamma \sin\beta +\sin\gamma \sin\alpha \cos\beta )\\ & -\Delta \alpha_{0}\sin\alpha \cos\beta -\Delta \theta_{om}\sin\beta +\Delta \theta_{im}\sin\alpha \sin\beta +(M_{azy}-\Delta \eta_{x})\sin\alpha -(M_{azx}+\Delta \beta_{0}+\Delta \eta_{y})\cos\alpha \sin\beta \end{array}\right.$$

(11) $$\left\{ \begin{array}{ll} N_{gx}= & -(1+\Delta K_{gx}+\omega_{e}\sin L)\cos\alpha \sin\beta +(\Delta \alpha_{0}+\omega_{e}\cos L)\sin\alpha \sin\beta -\Delta \theta_{om}\cos\beta +\\ & (\Delta \eta_{z}+M_{gxy})\sin\alpha +(M_{gxz}-\Delta \beta_{0}-\Delta \eta_{y})\cos\alpha \cos\beta +\Delta \theta_{im}\sin\alpha \cos\beta +b_{gx}\\ N_{gy}= & (1+\Delta K_{gy}+\omega_{e}\sin L)\sin\alpha +(\Delta \alpha_{0}+\omega_{e}\cos L)\cos\alpha +(\Delta \eta_{z}-M_{gyx})\cos\alpha \sin\beta +\\ & (M_{gyz}+\Delta \eta_{x})\cos\alpha \cos\beta +b_{gy}\\ N_{gz}= & (1+\Delta K_{gz}+\omega_{e}\sin L)\cos\alpha \cos\beta -(\Delta \alpha_{0}+\omega_{e}\cos L)\sin\alpha \cos\beta -\Delta \theta_{om}\sin\beta +\\ & (M_{gzy}-\Delta \eta_{x})\sin\alpha -(M_{gzx}+\Delta \beta_{0}+\Delta \eta_{y})\cos\alpha \sin\beta +\Delta \theta_{im}\sin\alpha \sin\beta +b_{gz} \end{array}\right.$$

From Equations (10) and (11), it can be seen that the output pulses of the accelerometers and the gyros are a function of their respective scale factor errors, biases, mounting errors and turntable errors.

To facilitate data processing, the Equations (10) and (11) are generally written in the form of a matrix. Error model for accelerometers in matrix form is:

(12) $$N^{a}=\Phi^{a}K^{a}$$

Defined the coefficient vector as $K^{a}={\left[\begin{array}{cccc} b_{a}^{T} & \Delta K_{a}^{T} & \Delta A^{T} & \Delta B^{T} \end{array}\right]}_{17\times 1}^{T}$

The accelerometer’s output vector is $N^{a}={\left[\begin{array}{ccc} \begin{array}{ccc} \frac{N_{ax_{1}}}{K_{ax}} & \frac{N_{ay_{1}}}{K_{ay}} & \frac{N_{az_{1}}}{K_{az}} \end{array} & \cdots & \begin{array}{ccc} \frac{N_{ax_{n}}}{K_{ax}} & \frac{N_{ay_{n}}}{K_{ay}} & \frac{N_{az_{n}}}{K_{az}} \end{array} \end{array}\right]}_{3n\times 1}^{T}$;

The structural matrix is $\Phi^{a}={\left[\begin{array}{cccc} I_{3\times 3} & C_{1}^{a} & D_{1}^{a} & E_{1}^{a}\\ \vdots & \vdots & \vdots & \vdots \\ I_{3\times 3} & C_{n}^{a} & D_{n}^{a} & E_{n}^{a} \end{array}\right]}_{3n\times 17}$; where $b_{a}={\left[\begin{array}{ccc} b_{ax} & b_{ay} & b_{ay} \end{array}\right]}_{3\times 1}^{T}$; $\Delta K_{a}={\left[\begin{array}{ccc} 1+\Delta K_{ax} & 1+\Delta K_{ay} & 1+\Delta K_{az} \end{array}\right]}_{3\times 1}^{T}$; $C_{i}^{a}={\left[\begin{array}{ccc} -\cos\alpha_{i}\sin\beta_{i} & 0 & 0\\ 0 & \sin\alpha_{i} & 0\\ 0 & 0 & \cos\alpha_{i}\cos\beta_{i} \end{array}\right]}_{3\times 3}$;

$$\Delta A={\left[\begin{array}{cccccc} \Delta \theta_{x0} & \Delta \theta_{y0} & \Delta \eta_{z} & \Delta \alpha_{0} & -(\Delta \beta_{0}+\Delta \eta_{y}) & -\Delta \theta_{om} \end{array}\right]}_{6\times 1}^{T}$$

$$\Delta B={\left[\begin{array}{ccccc} \Delta \theta_{im} & -M_{ayx}+\Delta \eta_{z} & M_{azy}-\Delta \eta_{x} & -M_{azx}-(\Delta \beta_{0}+\Delta \eta_{y}) & \Delta \eta_{x} \end{array}\right]}_{5\times 1}^{T}$$

$$D_{i}^{a}={\left[\begin{array}{cccccc} \sin\gamma_{i}\cos\beta_{i}+\cos\gamma_{i}\sin\alpha_{i}\sin\beta_{i} & -\cos\gamma_{i}\cos\beta_{i}+\sin\gamma_{i}\sin\alpha_{i}\sin\beta_{i} & \sin\alpha_{i} & \sin\alpha_{i}\sin\beta_{i} & \cos\alpha_{i}\cos\beta_{i} & \cos\beta_{i}\\ \cos\gamma_{i}\cos\alpha_{i} & \sin\gamma_{i}\cos\alpha_{i} & 0 & \cos\alpha_{i} & 0 & 0\\ \sin\gamma_{i}\sin\beta_{i}-\cos\gamma_{i}\sin\alpha_{i}\cos\beta_{i} & -(\cos\gamma_{i}\sin\beta_{i}+\sin\gamma_{i}\sin\alpha_{i}\cos\beta_{i}) & 0 & -\sin\alpha_{i}\cos\beta_{i} & 0 & \sin\beta_{i} \end{array}\right]}_{3\times 6}$$

$$E_{i}^{a}={\left[\begin{array}{ccccc} \sin\alpha_{i}\cos\beta_{i} & 0 & 0 & 0 & 0\\ 0 & \cos\alpha_{i}\sin\beta_{i} & 0 & 0 & \cos\alpha_{i}\cos\beta_{i}\\ \sin\alpha_{i}\sin\beta_{i} & 0 & \sin\alpha_{i} & \cos\alpha_{i}\sin\beta_{i} & 0 \end{array}\right]}_{3\times 5}$$

Similarly, the matrix form of the gyros model is:

(13) $$N^{g}=\Phi^{g}K^{g}$$

The coefficient vector to be identified is $K^{g}={\left[\begin{array}{cccc} b_{g}^{T} & \Delta F^{T} & \Delta M^{T} & \Delta H^{T} \end{array}\right]}_{15\times 1}^{T}$

The gyro’s output vector is:

$$N^{g}={\left[\begin{array}{ccc} \begin{array}{ccc} \frac{\sum_{0}^{t}N_{gx_{1}}}{K_{gx}} & \frac{\sum_{0}^{t}N_{gy_{1}}}{K_{gy}} & \frac{\sum_{0}^{t}N_{gz_{1}}}{K_{gz}} \end{array} & \cdots & \begin{array}{ccc} \frac{\sum_{0}^{t}N_{gx_{n}}}{K_{gx}} & \frac{\sum_{0}^{t}N_{gy_{n}}}{K_{gy}} & \frac{\sum_{0}^{t}N_{gz_{n}}}{K_{gz}} \end{array} \end{array}\right]}_{3n\times 1}^{T}$$

The structural matrix is:

$$\Phi^{g}={\left[\begin{array}{cccc} tI_{3\times 3} & 2\pi C_{1}^{g} & 2\pi D_{1}^{g} & 2\pi E_{1}^{g}\\ \vdots & \vdots & \vdots & \vdots \\ tI_{3\times 3} & 2\pi C_{n}^{g} & 2\pi D_{n}^{g} & 2\pi E_{n}^{g} \end{array}\right]}_{3n\times 15}$$

where $b_{g}={\left[\begin{array}{ccc} b_{gx} & b_{gy} & b_{gy} \end{array}\right]}_{3\times 1}^{T}$; $\Delta F=\Delta K_{g}+W_{e}$; $\Delta K_{g}={\left[\begin{array}{ccc} 1+\Delta K_{gx} & 1+\Delta K_{gy} & \begin{array}{c} 1+\Delta K_{gz} \end{array} \end{array}\right]}_{3\times 1}^{T}$;

$$W_{e}=\omega_{e}\sin L\cdot t/2\pi \cdot {\left[\begin{array}{ccc} 1 & 1 & 1 \end{array}\right]}_{3\times 1}^{T}$$

$$\Delta M={\left[\begin{array}{cccc} M_{gxy}+\Delta \eta_{z} & M_{gxz}-(\Delta \beta_{0}+\Delta \eta_{y}) & -M_{g}{}_{yx}+\Delta \eta_{z} & M_{gyz}+\Delta \eta_{x} \end{array}\right]}_{4\times 1}^{T}$$

$$\Delta H={\left[\begin{array}{ccccc} M_{gzy}-\Delta \eta_{x} & -(M_{gzx}+\Delta \beta_{0}+\Delta \eta_{y}) & \Delta \alpha_{0}+\omega_{e}\sin L\cdot t/2\pi & -\Delta \theta_{om} & \Delta \theta_{im} \end{array}\right]}_{5\times 1}^{T}$$

$$C_{i}^{g}={\left[\begin{array}{ccc} -\cos\alpha_{i}\sin\beta_{i} & 0 & 0\\ 0 & \sin\alpha_{i} & 0\\ 0 & 0 & \cos\alpha_{i}\cos\beta_{i} \end{array}\right]}_{3\times 3}$$

$$D_{i}^{g}={\left[\begin{array}{cccc} \sin\alpha_{i} & \cos\alpha_{i}\cos\beta_{i} & 0 & 0\\ 0 & 0 & \cos\alpha_{i}\sin\beta_{i} & \cos\alpha_{i}\cos\beta_{i}\\ 0 & 0 & 0 & 0 \end{array}\right]}_{3\times 4}$$

$$E_{i}^{g}={\left[\begin{array}{ccccc} 0 & 0 & \sin\alpha_{i}\sin\beta_{i} & \cos\beta_{i} & \sin\alpha_{i}\cos\beta_{i}\\ 0 & 0 & \cos\alpha_{i} & 0 & 0\\ \sin\alpha_{i} & \cos\alpha_{i}\sin\beta_{i} & -\sin\alpha_{i}\cos\beta_{i} & \sin\beta_{i} & \sin\alpha_{i}\sin\beta_{i} \end{array}\right]}_{3\times 5}$$

Aiming at Equations (12) and (13), the coefficient vectors of the accelerometers and gyros can be identified by the Least Square method, the coefficient vectors of the accelerometer and the gyro can be obtained:

(14) $$K^{a}={\left(\Phi^{a}{}^{T}\Phi^{a}\right)}^{-1}\Phi^{a}{}^{T}N^{a}$$

(15) $$K^{g}={\left(\Phi^{g}{}^{T}\Phi^{g}\right)}^{-1}\Phi^{g}{}^{T}N^{g}$$

With residual error vectors $\varepsilon_{a}=N^{a}-\Phi^{a}K^{a}$ and $\varepsilon_{g}=N^{g}-\Phi^{g}K^{g}$, we obtain the unitary weight standard deviations of the accelerometers’ and the gyros’ outputs:

(16) $$\sigma_{a}=\sqrt{\frac{\varepsilon_{a}{}^{T}\varepsilon_{a}}{q-m}}$$

(17) $$\sigma_{g}=\sqrt{\frac{\varepsilon_{g}{}^{T}\varepsilon_{g}}{q-m}}$$

where *q* is the total number of samples, and *m* is the number of identified coefficients. It can easily be seen that the scale factors, biases of the accelerometers’ and the gyros’ are obtained from the first six elements of $K^{a}$ and $K^{g}$. After algebraic operations of the elements in $K^{a}$ and $K^{g}$, the installation errors of the accelerometer and the gyro, the turntable errors are derived as follows:

(18) $$\left\{ \begin{array}{l} M_{ayx}=-K^{a}(14)+K^{a}(9);M_{azx}=K^{a}(11)-K^{a}(16)\\ M_{azy}=K^{a}(15)+K^{a}(17);\Delta \theta_{x0}=K^{a}(7);\Delta \theta_{y0}=K^{a}(8)\\ \Delta \theta_{om}=-K^{a}(12);\Delta \theta_{im}=K^{a}(13);\Delta \eta_{x}=K^{a}(17)\\ \Delta \beta_{0}+\Delta \eta_{y}=-K^{a}(11);\Delta \eta_{z}=K^{a}(9);\Delta \alpha_{0}=K^{a}(10) \end{array}\right.$$

(19) $$\left\{ \begin{array}{l} M_{gxy}=K^{g}(7)-\Delta \eta_{z}=K^{g}(7)-K^{a}(9)\\ M_{gxz}=K^{g}(8)+(\Delta \beta_{0}+\Delta \eta_{y})=K^{g}(8)-K^{a}(11)\\ M_{gyx}=-K^{g}(9)+\Delta \eta_{z}=-K^{g}(9)+K^{a}(9)\\ M_{gyz}=K^{g}(10)-\Delta \eta_{x}=K^{g}(10)-K^{a}(17)\\ M_{gzx}=-K^{g}(12)-(\Delta \beta_{0}+\Delta \eta_{y})=-K^{g}(12)+K^{a}(11)\\ M_{gzy}=K^{g}(11)+\Delta \eta_{x}=K^{g}(11)+K^{a}(17) \end{array}\right.$$

where $K^{a}(i)$ and $K^{g}(i)$ are the *i*^th^ elements of vector $K^{a}$ and $K^{g}$ respectively.

## 3. Calibration Scheme

For multi-position calibration of an IMU on a turntable, the norm of the angular rates and of the specific forces measured by the accelerometers and the gyros respectively are equal to the angular rates and the specific forces uniform distribution produced by the gravity, and angular rate produced by outer axis of the turntable, which means that the input components of the accelerometers and the gyros are distributed on spherical surfaces.

The uniform distribution of input components on the spherical surface, i.e., the test points for accelerometer and gyro calibration being on the spherical surfaces, make the calibration scheme comply with the D-suboptimal test plan criterion, minimize the number of descending rank of the information matrix and simultaneously maximize the amount of test information, and greatly raise the calibration accuracy of model parameters.

To make a calibration scheme with a uniform distribution of test points on the sphere is to seek the apices of the polyhedron. In this study, a regular dodecahedron 20-point scheme is chosen. The center of the regular dodecahedron is the body frame’s origin. The outer axis of the turntable is perpendicular to one of the plane of the regular dodecahedron. The middle and the inner axes rotate to the angles in Table 2. Then the test points can be uniformly distributed on the vertices of the regular dodecahedron: {(0,±1/ϕ,±ϕ), (±1/ϕ,±ϕ,0), (±ϕ,0,±1/ϕ), (±1,±1,±1)}, where $\phi =(-1+\sqrt{5})/2$, a D-suboptimal calibration scheme is realized.

## 4. Analysis of Simulation Results

The simulation was performed under the following conditions: the local latitude N-45.73265°, the outer axis rotates anticlockwise about 360° at angular rate of 10°/s, the random errors of the accelerometers in IMU are all 10 μg (1σ), and the random errors of the gyros are all 0.01°/h (1σ); the accelerometers’ scale factors are 1.082 × 10^4^p/(s·g), 1.038 × 10^4^p/(s·g), 1.058 × 10^4^p/(s·g), and the scale factors of gyros are all 4.272 × 10^3^′′/p, the turntable errors Δ*η_x_*_4_, Δ*η_y_*_4_, Δ*η_z_*_4_, Δ*θ_x_*_0_, Δ*θ_y_*_0_, Δ*θ_om_*, Δ*θ_im_,* Δ*α*_0_, and Δ*β*_0_ are all 1′.

For the convenience of comparison, simulations were performed with two sets of data: data without turntable errors and data with turntable errors. According to the two sets of data, the IMU parameters were calibrated respectively using the new model and the traditional one established in this paper. Table 3 shows the simulation preset values of model parameters in the IMU and the calibration errors (i.e., calibration error = simulation preset value − calibration result).

Table 3 shows that: when there are no turntable errors, the calibration results of the two methods are almost the same, and the calibration errors of parameters are of the same order of magnitude. When turntable errors are considered, the calibration errors of model parameters using the traditional model are obviously larger by one order of magnitude than those produced using the new one. For comparison, the calibration results of the error parameters of the new model are not affected by turntable errors. The calibration errors of model parameters are still in the same order of magnitude as the calibration results obtained without turntable errors.

**Figure 1:**
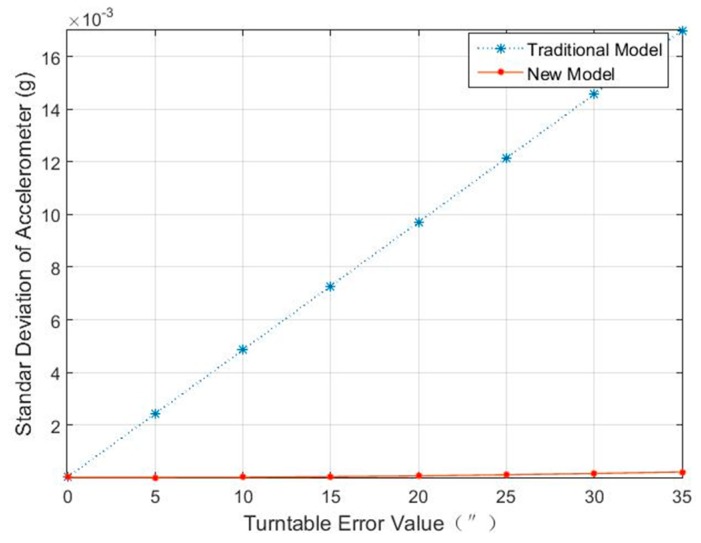
Unitary weight standard deviations of accelerometer calibrated in turntable with different errors.

**Figure 2:**
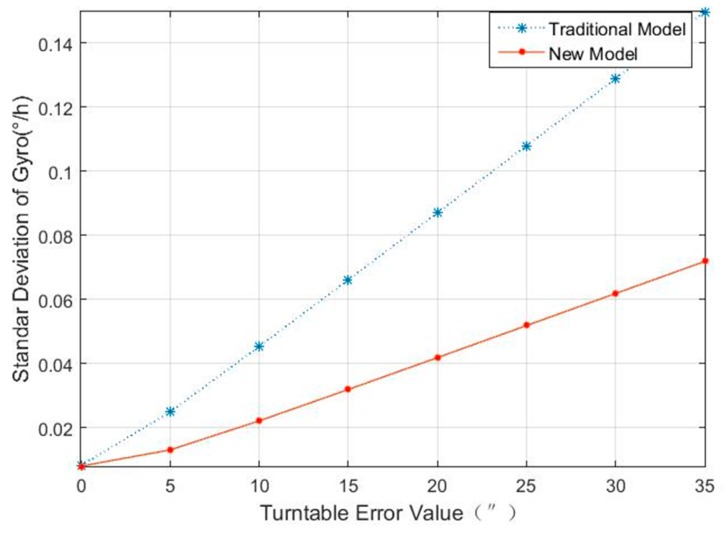
Unitary weight standard deviations of gyro in different turntable errors.

To clearly show the calibration accuracy, the unitary weight standard deviations of the accelerometers’ and gyros’ (i.e., $\sigma_{a}$ and $\sigma_{g}$) were calculated using the formulas in Section 2, through the data of the accelerometer and the gyro in 20 static positions. Figure 1 and Figure 2 show the corresponding standard deviations for the accelerometer and the gyro under different turntable errors.

Comparing the unitary weight standard deviations of the accelerometers’ and the gyros’ in Figure 1 and Figure 2, it can be seen that the traditional model is greatly influenced by turntable errors, while the new model is little affected by turntable errors. Under the traditional model, when the turntable error increased to 35′, the unitary weight standard deviations of the accelerometers’ and the gyros’ were 1507 times and 18 times larger than that in the condition where there were no turntable errors; while under the new model, the accelerometers’ and the gyros’ unitary weight were only 19 times and 9 times larger. When the turntable error increased to 5′, the unitary weight standard deviations of the gyros’ under the new model was 0.0131°/h, which was equivalent to the gyro noise, but the unitary weight standard deviations of the gyros′ had increased to 0.0249°/h under the traditional model. At the same time, when the turntable error increased to 10′, the unitary weight standard deviations of the accelerometers’ under the new model were all 1.79 × 10^−5^ g, which were still in the same order of magnitude with their noise, but the unitary weight standard deviations of the accelerometers’ had increased to 4.85 × 10^−5^ g. To examine the new model’s accuracy for turntable error identification, Table 4 shows the calibration errors for turntable error in the new model with different turntable errors.

From Table 4, it can be seen that, though the calibration errors for turntable errors tend to increase with the increase of turntable errors, the maximum calibration error is only 2.8% of the simulation preset value. As the turntable error increased to 35′, the second-order small quantity neglected in the previous formula had reached 1.04 × 10^−4^ rad (about 21′′), leading to the increase of calibration error for the turntable error to 59′′. From the engineering meaning, it is easy to control the accuracy of the turntable within 10′, which shows that in practical applications, the results of turntable error identification obtained with the new model can be very close to the simulation preset values, achieving an arc second level of identification accuracy. The above simulation results verified the correctness of establishing calibration model considering the turntable errors, so that the calibration results of the model parameters are closer to the simulation preset values. It not only effectively avoided the influence of turntable errors on the IMU calibration accuracy, but also accurately identified the turntable errors.

## 5. Model Validation Experiment and Analysis

To conveniently determine the impact of the turntable errors on IMU calibration accuracy, turntable errors were introduced in the IMU calibration experiment. Since the perpendicularity in the three-axis turntable cannot be modified because of turntable’s mechanical structure, we can deliberately change the turntable error sources such as the zero position errors, the mounting errors of the IMU on the turntable, and the outer axis perpendicularity with respect to horizontal plane. Among them, the zero position errors are the most controllable. In this experiment, zero position error −5′ was introduced to the middle axis of the three-axis turntable to verify the practicability of the model.

**Figure 3:**
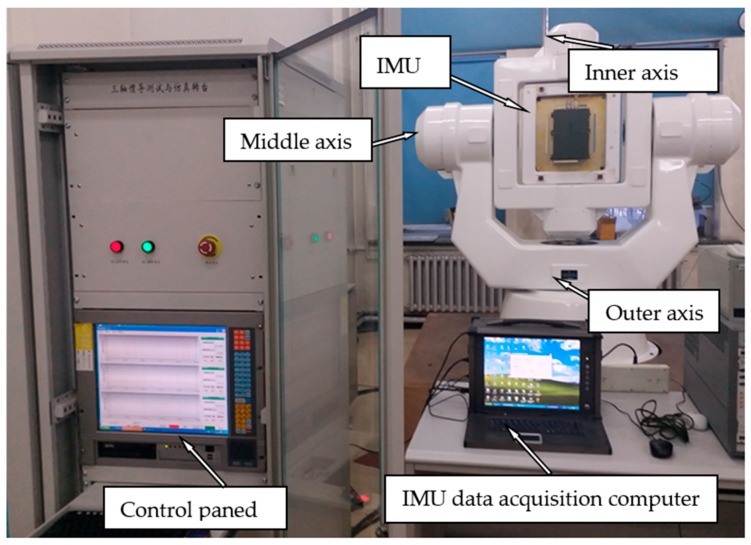
IMU calibration experiment on high-precision three-axis turntable.

For calibration experiment on a certain type of laser gyro strapdown inertial navigation system (Figure 3), the gyro’s measurement accuracy in INS is higher than 0.01°/h, the accelerometer’s measurement accuracy is higher than 1 × 10^−4^ g, the perpendicularities and angular position errors of the turntable are all less than 1′′ (1σ). The calibration scheme adopts the D-suboptimal test plan, where there are 20 positions for calibration of accelerometer data, and the time in each static position is 121 s. For gyro calibration, when the gyro rotates 360° at the angular rate of 10°/s around the outer axis of the turntable, the inner axis and middle axis are in the corresponding angular position and attitude of the 20-point test plan. The time for data collection at each position is 40 s; the gyro bias was measured using a two-position method, and the time for data collection at each position is 30 min.

By comparison, the experimental data before and after the introduction of the zero position errors of the turntable’s middle axis are used for calibration in the new model and the traditional model. Table 5 shows the calibration results and deviations of the two models before and after the introduction of the zero position errors of middle axis.

From Table 5, it can be seen that the calibration results of the new model before and after the introduction of the middle axis zero position errors in the turntable are nearly the same, but the calibration error of the model parameters obviously increases with the introduction of the turntable’s zero position errors in the traditional model. This shows that the traditional model is greatly affected by turntable errors, while the new model can effectively inhibit the influence of turntable errors on the calibration accuracy of IMU parameters.

At the same time, using data of the accelerometers’ and the gyros’ from 20 static positions in the D−suboptimal test plan, Table 6 gives the unitary weight standard deviations of the accelerometers’ and the gyros’ before and after the introduction of the zero position errors of the turntable axis (i.e., *σ_a_* and *σ_g_*).

Comparison of the data in Table 6 shows that for calibration under the traditional model, with the introduction of zero position errors of the turntable’s middle axis, the unitary weight standard deviations of the gyros’ increase by 0.002 °/h, and unitary weight standard deviations of the accelerometers’ increase 1.5 times. However, the unitary weight standard deviations of the accelerometers’ and the gyros’ are not affected by turntable errors; the unitary weight standard deviations obtained after introducing zero position errors are in the same order of magnitude as before introducing zero position errors.

From the above analysis, we can see that turntable errors have a great influence on the calibration accuracy of the IMU parameters under the traditional model, but the calibration results of the IMU parameters under the new model are not affected by them. In practical applications, adopting the new model can effectively improve IMU calibration accuracy, and at the same time turntable errors are identified and automatically compensated.

## 6. Conclusions

This paper studies an IMU calibration method for overcoming the impacts of turntable errors, including the axis perpendicularities, zero position errors, mounting errors between the IMU and the turntable, on IMU calibration accuracy.

By establishing the relationship between turntable errors and the calibration errors of IMU model parameters, turntable errors are introduced to the IMU calibration model parameters, so the output of the IMU is a function of the scale factor errors, biases, mounting errors, and turntable errors.

Based on the D-suboptimal design, a new calibration scheme was designed using a regular dodecahedron-20 with 20 points uniformly distributed on the sphere.

The proposed calibration method can effectively identify turntable errors and the IMU error model parameters simultaneously, and can avoid the influence of turntable errors on IMU calibration accuracy.

The simulation analysis shows that the calibration accuracy of turntable errors obtained by this method is of an arcsecond level, and the calibration error is less than 2.8%; when turntable errors are all 1′, the IMU model parameter errors obtained by this method are reduced by an order of magnitude compared with the traditional method, and are closer to the simulation preset values. The results of the theoretical analysis are verified experimentally, and the two sets of data before and after introducing of turntable errors are used as comparison. With the introduction of turntable errors, the IMU calibration accuracy with the traditional method is obviously reduced. The calibration method proposed in this paper can effectively restrain the impact of turntable errors, and make IMU calibration accuracy before and after introducing turntable error be in the same order of magnitude.

The method proposed in this paper has the advantages of overcoming the dependence on the turntable’s precision in the calibration process, reducing the calibration cost, and some of turntable errors are automatically compensated, and the calibration accuracy is raised greatly.

## Figures and Tables

**Table 1:** Frame definition.

Frame	Description
*n*	The navigation frame
*t*1	The outer axis frame
*t*2	The middle axis frame
*t*3	The inner axis frame
*b*	The body frame
*a*	Accelerometers frame
*g*	Gyros frame

**Table 2:** The uniformly distributed positions.

	**1**	**2**	**3**	**4**	**5**	**6**	**7**	**8**	**9**	**10**
*α*	35.2644°	35.2644°	324.7356°	215.2644°	144.7356°	144.7356°	324.7356°	324.7356°	69.0948°	69.0948°
*β*	315°	225°	315°	45°	225°	315°	45°	135°	0°	180°
*γ*	18°	36°	54°	72°	90°	108°	126°	144°	162°	180°
	**11**	**12**	**13**	**14**	**15**	**16**	**17**	**18**	**19**	**20**
*α*	290.9052°	290.9052°	20.9052°	399.0948°	20.9052°	339.0948°	0°	0°	0°	0°
*β*	0°	180°	270°	90°	90°	90°	339.0948°	200.9052°	20.9052°	159.0948°
*γ*	198°	216°	234°	252°	270°	288°	306°	324°	342°	360°

**Table 3:** Results estimated in two models.

IMU Parameters	Preset Values of SIMULATION	Errors Calibrated by New Model		Errors Calibrated by Traditional Model	
		Without Turntable Errors	With Turntable Errors	Without Turntable Errors	With Turntable Errors
Δ*K_ax_* (ppm)	700	2.118	2.475	1.860	25.925
Δ*K_ay_* (ppm)	800	−8.060	−8.034	−7.755	20.389
Δ*K_az_* (ppm)	500	−0.850	−1.683	−1.458	−48.233
*b_ax_* (μg)	700	0.702	0.659	0.447	80.308
*b_ay_* (μg)	−900	4.259	4.205	6.328	−119.587
*b_az_* (μg)	−3000	3.703	3.717	3.994	3.234
*M_ayx_* (’)	−1	−0.0094	−0.0105	0.0074	0.5738
*M_azx_* (’)	−5	0.0012	0.0017	0.0050	−3.5655
*M_azy_* (’)	−1	−0.0384	−0.0393	−0.0266	1.1399
Δ*K_gx_* (ppm)	−81	−0.160	1.3	−0.133	65.891
Δ*K_gy_* (ppm)	84	0.083	0.21	0.075	42.664
Δ*K_gz_* (ppm)	−22	−0.061	−5.1	−0.076	−81.947
*b_gx_* (°/*h*)	−0.7920	−0.0072	−0.0072	−0.0073	−0.0072
*b_gy_* (°/*h*)	0.1152	0.0029	0.0029	0.0029	0.0005
*b_gz_* (°/*h*)	0.5148	−0.0002	0.0030	0.0042	0.0074
*M_gxy_* (’)	5.00	−0.0003	−0.0005	−0.0004	−1.1834
*M_gxz_* (’)	−2.00	0.0000	−0.0012	0.0004	2.8588
*M_gyx_* (’)	0.35	−0.0001	−0.0007	−0.0001	0.9525
*M_gyz_* (’)	0.19	−0.0008	−0.0010	−0.0007	−1.1426
*M_gzx_* (’)	−17.00	0.0003	0.0035	0.0000	−2.8499
*M_gzy_* (’)	15.00	0.0001	0.0059	−0.0001	0.7203

**Table 4:** The Calibration errors for turntable errors.

Simulation Preset Value	Calibration Error							
	Δ*η_x_*	Δ*η_y_* + Δ*β*_0_	Δ*η_z_*	Δ*θ_x_*_0_	Δ*θ_y_*_0_	Δ*θ_om_*	Δ*θ_im_*	Δ*α*_0_
0′	1′′	1′′	1′′	−1′′	0′′	0′′	0′′	−1′′
5′	3′′	0′′	1′′	0′′	−1′′	0′′	1′′	0′′
10′	7′′	−2′′	2′′	3′′	−2′′	1′′	2′′	0′′
15′	13′′	−7′′	3′′	7′′	−5′′	3′′	4′′	0′′
20′	21″	−13′′	4′′	13′′	−8′′	5′′	8′′	−1′′
25′	31′′	−22′′	6′′	21′′	−13′′	8′′	12′′	−1′′
30′	44′′	−33′′	8′′	30′′	−19′′	12′′	17′′	−2′′
35′	59′′	−46′′	10′′	42′′	−26′′	16′′	23′′	−3′′

**Table 5:** The results of two calibration experiments.

IMU Parameters	Calibration error of New Model			Calibration Error of Traditional Model		
	After adding Zero Position Errors	Before Adding Zero Position Errors	Calibration Deviations	After Adding Zero Position Errors	Before Adding Zero Position Errors	Calibration Deviations
Δ*K_ax_* (ppm)	625.486	628.362	−2.876	505.007	466.179	38.828
Δ*K_ay_* (ppm)	690.762	675.280	15.482	683.009	894.689	−211.680
Δ*K_az_* (ppm)	705.718	714.035	−8.316	703.488	600.453	103.035
*b_ax_* (μg)	689.206	687.529	1.677	680.151	906.562	−226.411
*b_ay_* (μg)	−990.669	−1019.763	29.094	−994.195	−1786.238	792.043
*b_az_* (μg)	−3246.494	−3242.265	−4.230	−3253.249	−3324.693	71.444
*M_ayx_* (′′)	−1.2637	−1.2299	−0.0338	3.1341	2.9001	0.2339
*M_azx_* (′′)	−4.0168	−4.0198	0.0031	−2.3224	−2.7854	0.4629
*M_azy_* (′′)	−0.2693	−0.2981	0.0287	−3.6184	−3.2182	−0.4002
Δ*K_gx_* (ppm)	−92.779	−93.685	0.906	−82.300	−126.587	44.288
Δ*K_gy_* (ppm)	78.956	78.112	0.844	33.551	244.984	−211.433
Δ*K_gz_* (ppm)	−50.020	−51.379	1.359	−28.749	−132.382	103.633
*b_gx_* (°/*h*)	−0.7951	−0.7951	0.0000	−0.7924	−0.7912	−0.0011
*b_gy_* (°/*h*)	0.1238	0.1233	0.0005	0.1105	0.1127	−0.0022
*b_gz_* (°/*h*)	0.3835	0.3837	−0.0002	0.3795	0.3806	−0.0010
*M_gxy_* (′′)	−3.9282	−3.9286	0.0003	1.2995	1.4764	−0.1769
*M_gxz_* (′′)	−3.1620	−3.1603	−0.0017	−3.9593	−4.3126	0.3533
*M_gyx_* (′′)	10.0201	10.0193	0.0007	4.8015	4.5670	0.2345
*M_gyz_* (′′)	−0.2913	−0.2922	0.0010	3.6240	2.9167	0.7073
*M_gzx_* (′′)	−16.9855	−16.9871	0.0016	−16.0115	−16.4744	0.4629
*M_gzy_* (′′)	15.8859	15.8867	−0.0008	12.0304	12.4323	−0.4019

**Table 6:** Standard deviation in two schemes.

Standard Deviation	New Model		Traditional Model	
	After Adding Zero Position Errors	Before Adding Zero Position Errors	After Adding Zero Position Errors	Before Adding Zero Position Errors
*σ_a_* (μg)	149.853	146.940	618.604	935.542
*σ_g_* (°/h)	0.0388	0.0390	0.0408	0.0420
